# Supplementary material for: Differential Response and Recovery Dynamics of HSPC Populations Following Plasmodium chabaudi Infection
Source: Int J Mol Sci. 2025 Mar 20;26(6):2816. doi: 10.3390/ijms26062816 (PMC11943058; doi:10.3390/ijms26062816)
Supplement: Supplementary file 1 [file ijms-26-02816-s001.zip › ijms-3369401-supplementary.pdf]

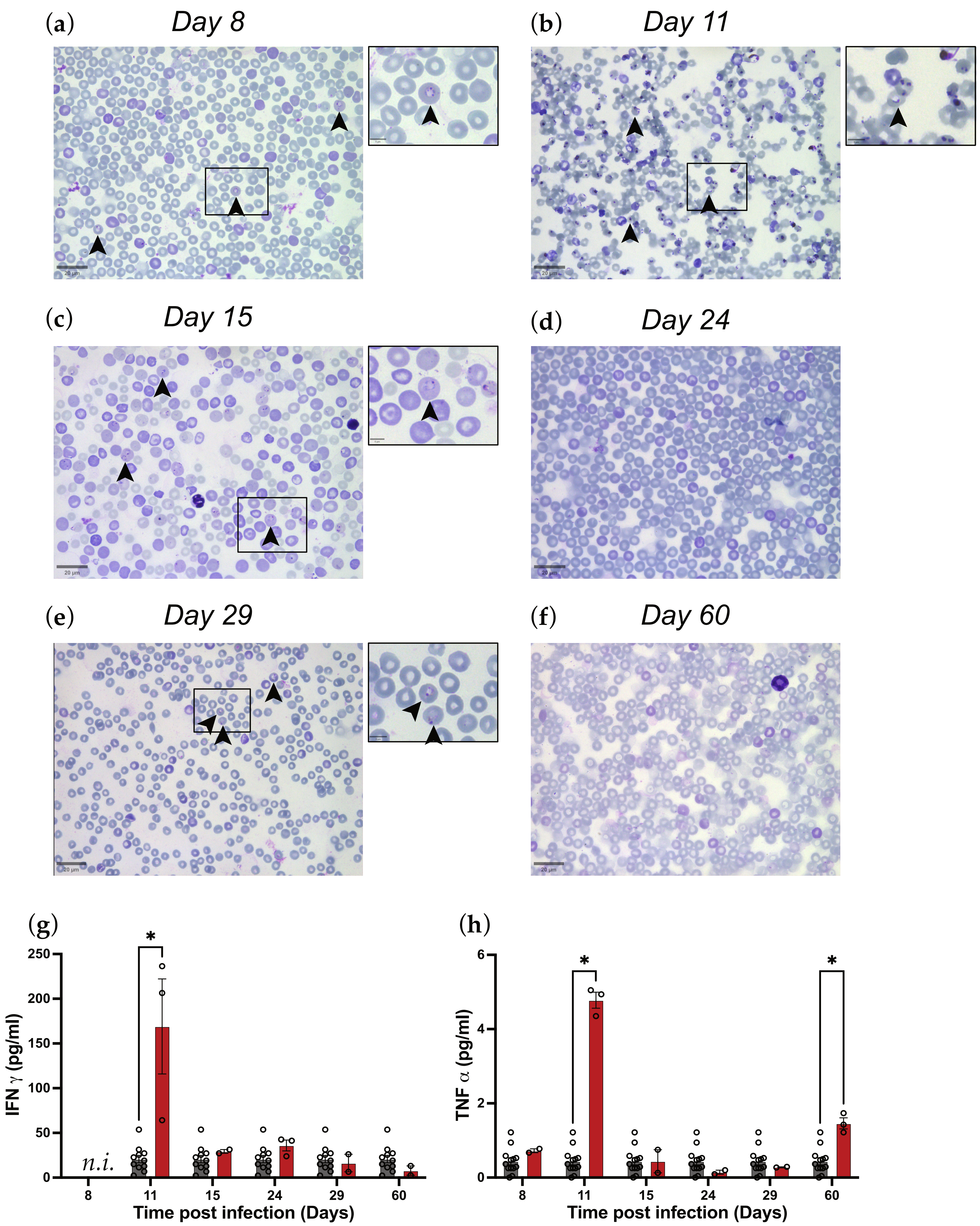

Figure S1. Parasitemia and cytokine levels throughout infection with *Plasmodium chabaudi*.

A-F. Representative Giemsa-stained blood smears collected from mice 8 (**A**), 11 (**B**), 15 (**C**), 24 (**D**), 29 (**E**) and 60 (**F**) days post infection. Arrows indicate examples of infected erythrocytes. **G-H**.Levels of IFN-γ (**G**), and TNF-α (**H**) in blood serum collected from infected and control mice at day 8, 11, 15, 24, 29 and 60 p.i. In **G-I** data are presented as mean +/- s.e.m. and p values (asterisks) were determined by unpaired two-tailed Student's *t*-test. * $p < 0.05$, ** $p < 0.01$, *** $p < 0.001$. n = 11-13 for control and n = 3 for infected mice at day 8, 11, 15, 24, 29, 60 p.i..

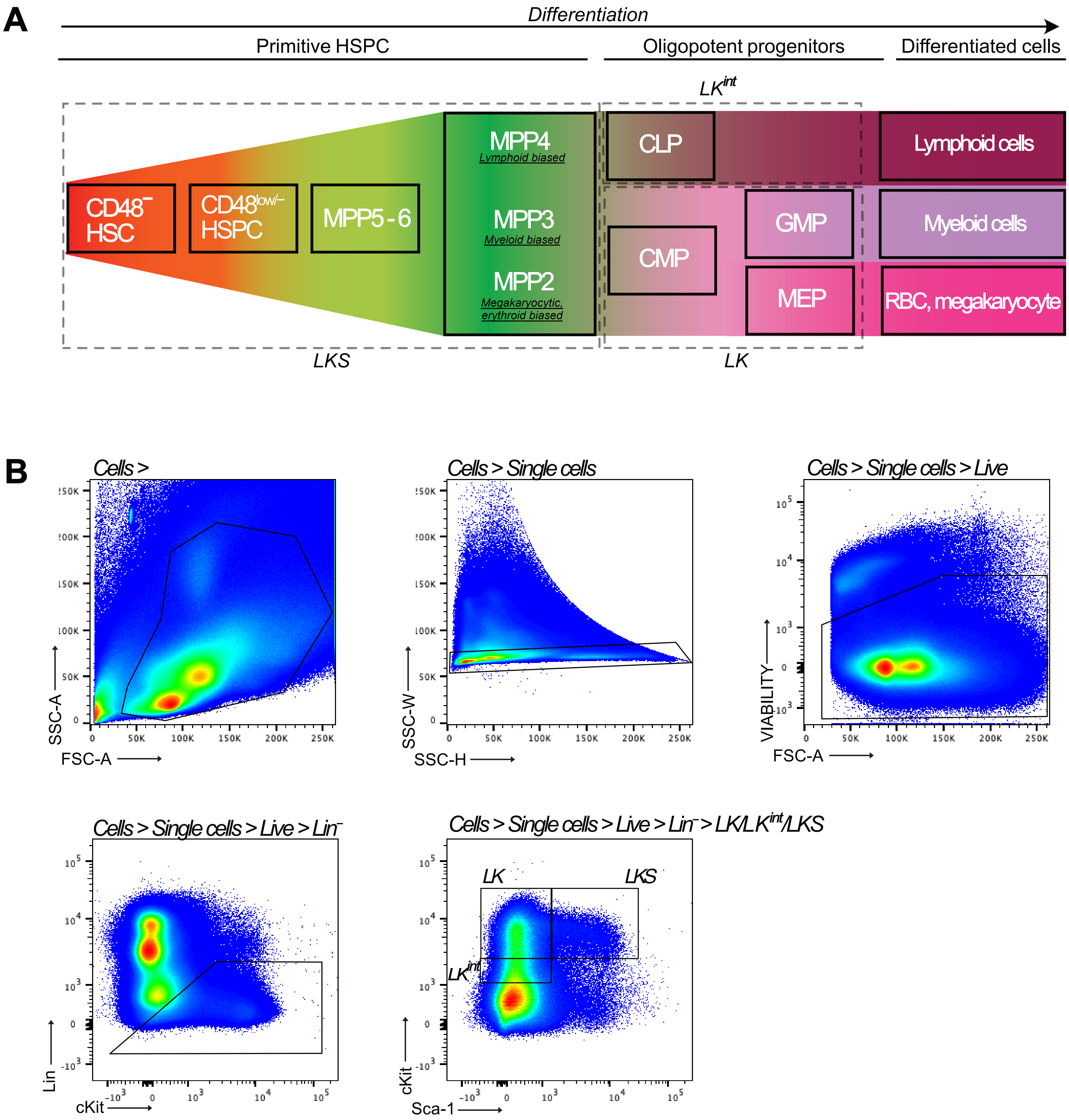

Figure S2. Hematopoietic populations investigated in response to *P. chabaudi* infection

A. Schematic representation summarizing the hierarchy of the different hematopoietic populations and the nomenclature used in this study. LKS cells include primitive HSPCs, thus HSCs and MPPs. Oligopotent progenitors are divided into LK and LK^{int} cells. LK cells include CMP upstream of GMP and MEP populations, and LK^{int} cells include CLPs. **B.** Flow cytometry plots showing the gating strategy used from all events to LK/LKS populations. The gating strategies for further subpopulations are shown in other figures.

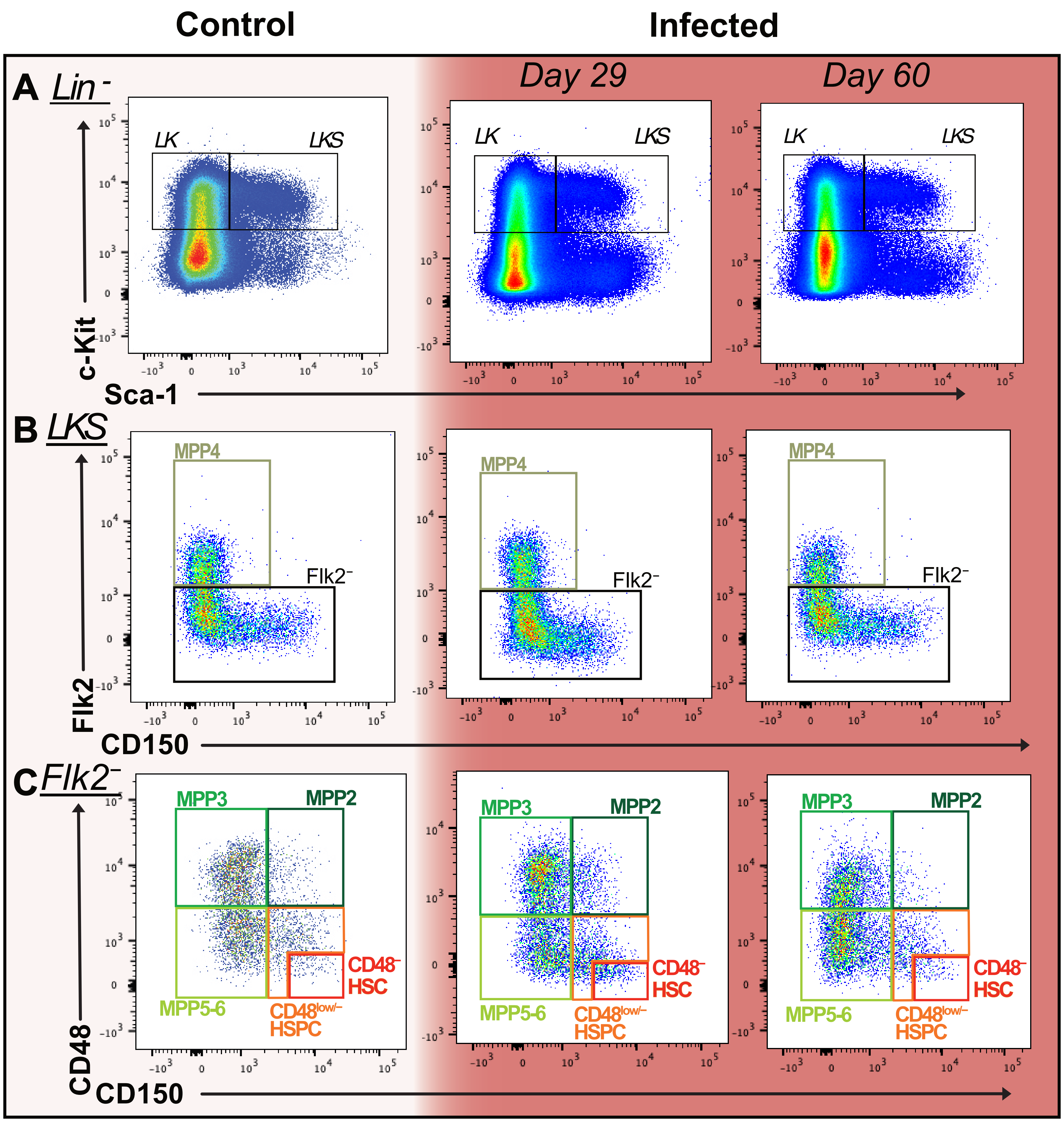

Figure S3. Primitive hematopoietic populations during the recovery phase of *P. chabaudi* infection.

A-E. Representative flow cytometry plots showing LK and LKS (**A**), MPP4 (**B**), MPP2, MPP3, MPP5-6, CD48$^-$ HSC and CD48$^{low/-}$ HSPC (**C**) populations in controls and infected mice at day 29 and day 60 p.i.

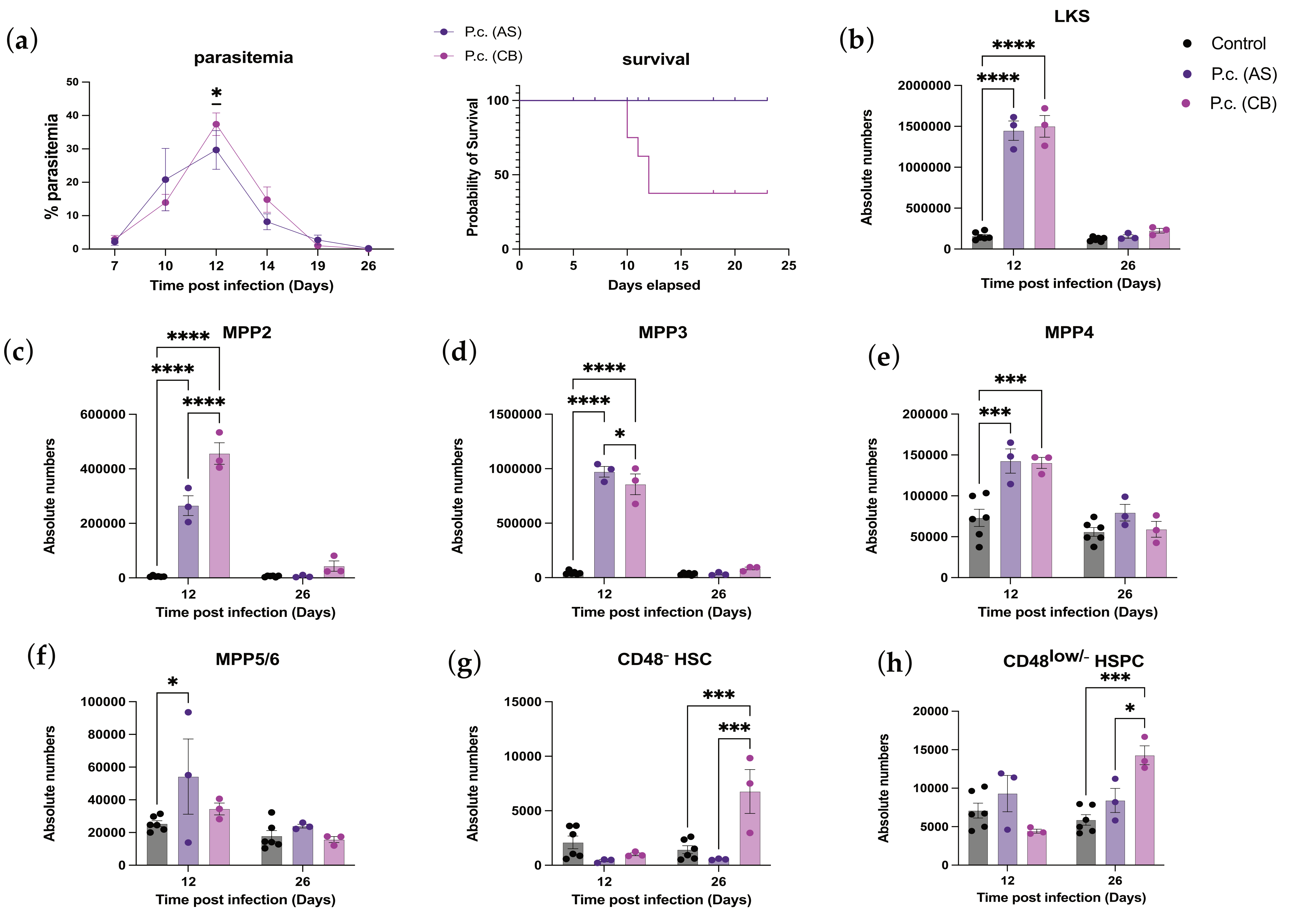

Figure S4. ***P. chabaudi CB* causes similar effects within the HSPC populations.**

A. At day 0, mice were either injected with *Plasmodium chabaudi AS or CB* infected RBCs, parasitemia and probability of survival were monitored throughout infection. BM analysis was conducted at day 12 (acute infection) and day 26 (recovery phase) post infection. **B-G.** Normalized absolute numbers of LKS (**B**), MPP2 (**C**), MPP3 (**D**), MPP4 (**E**), MPP5/6 (**F**), $CD48^-$ HSC (**G**) and $CD48^{low/-}$ HSPC (**H**) cell populations in control and infected mice at day 12 and 26 p.i.. In **B-H** data are presented as mean +/- s.e.m., and p values (asterisks) were determined by unpaired two-tailed Student's *t*-test. * $p < 0.05$, ** $p < 0.01$, *** $p < 0.001$. *n* = 6, 6, control and *n* = 3, 3 infected mice at day 12, 26 p.i. respectively. P.c., *Plasmodium chabaudi*, HSC, hematopoietic stem cell, HSPC, hematopoietic stem and progenitor cells, MPP, multipotent progenitor.

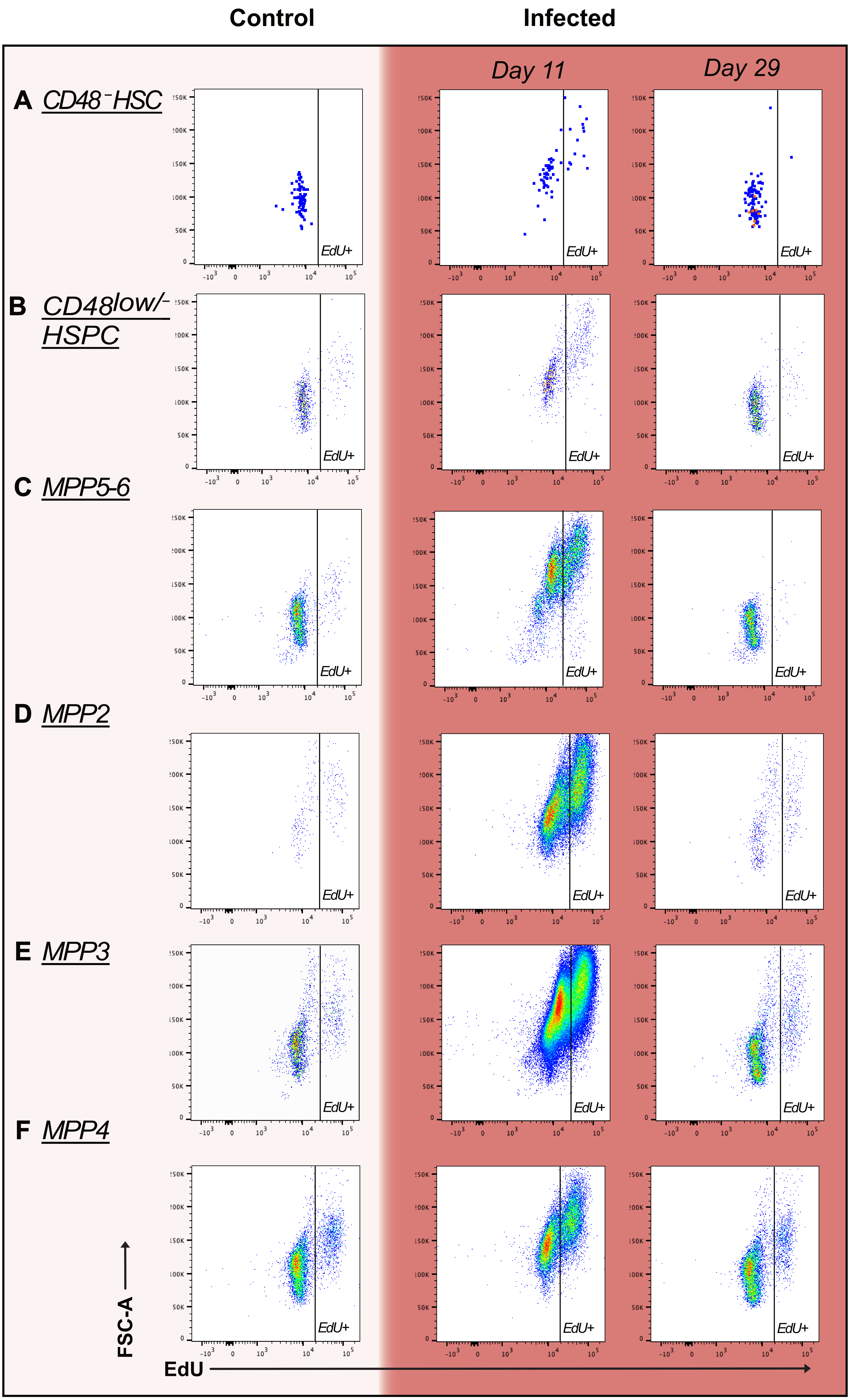

Figure S5. Proliferation of primitive hematopoietic populations during *P. chabaudi* infection.
A-E. Representative flow cytometry plots showing cells in the S-phase of the cell cycle (EdU^+) within $CD48^-$ HSC (**A**), $CD48^{low/-}$ HSPC (**B**), MPP5-6(**C**), MPP2 (**D**), MPP3 (**E**) and MPP4 (**F**) populations in controls and infected mice at day 11 and day 29 p.i.

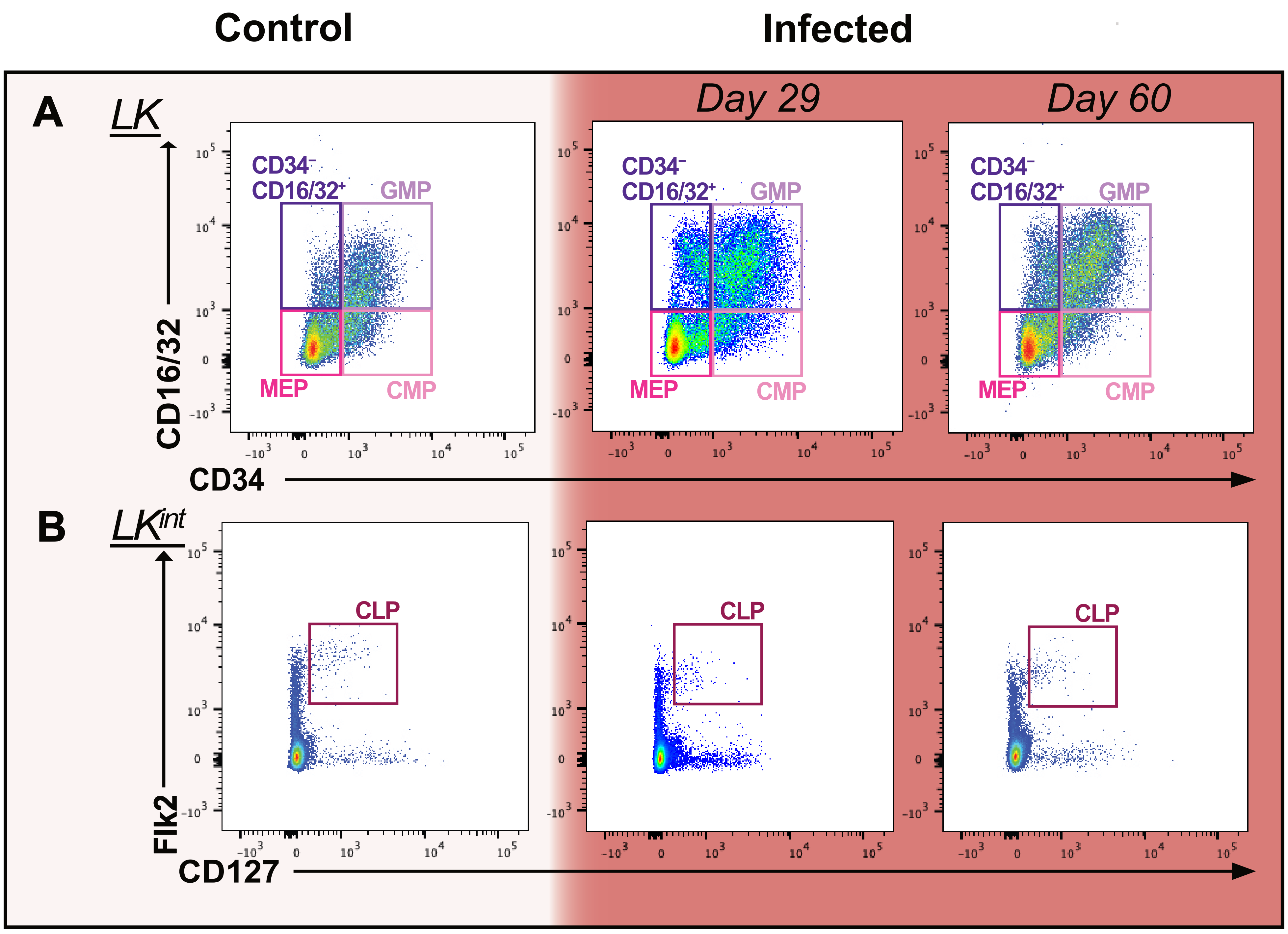

Figure S6. Hematopoietic populations during the recovery phase of *P. chabaudi* infection.

A-E. Representative flow cytometry plots showing (**A**) $CD34^{-}CD16/32^{+}$, GMP, CMP and MEP populations within the LK parent population and (**B**) CLP cells within the parent LK^{int} population in controls and infected mice at day 29 and day 60 p.i.

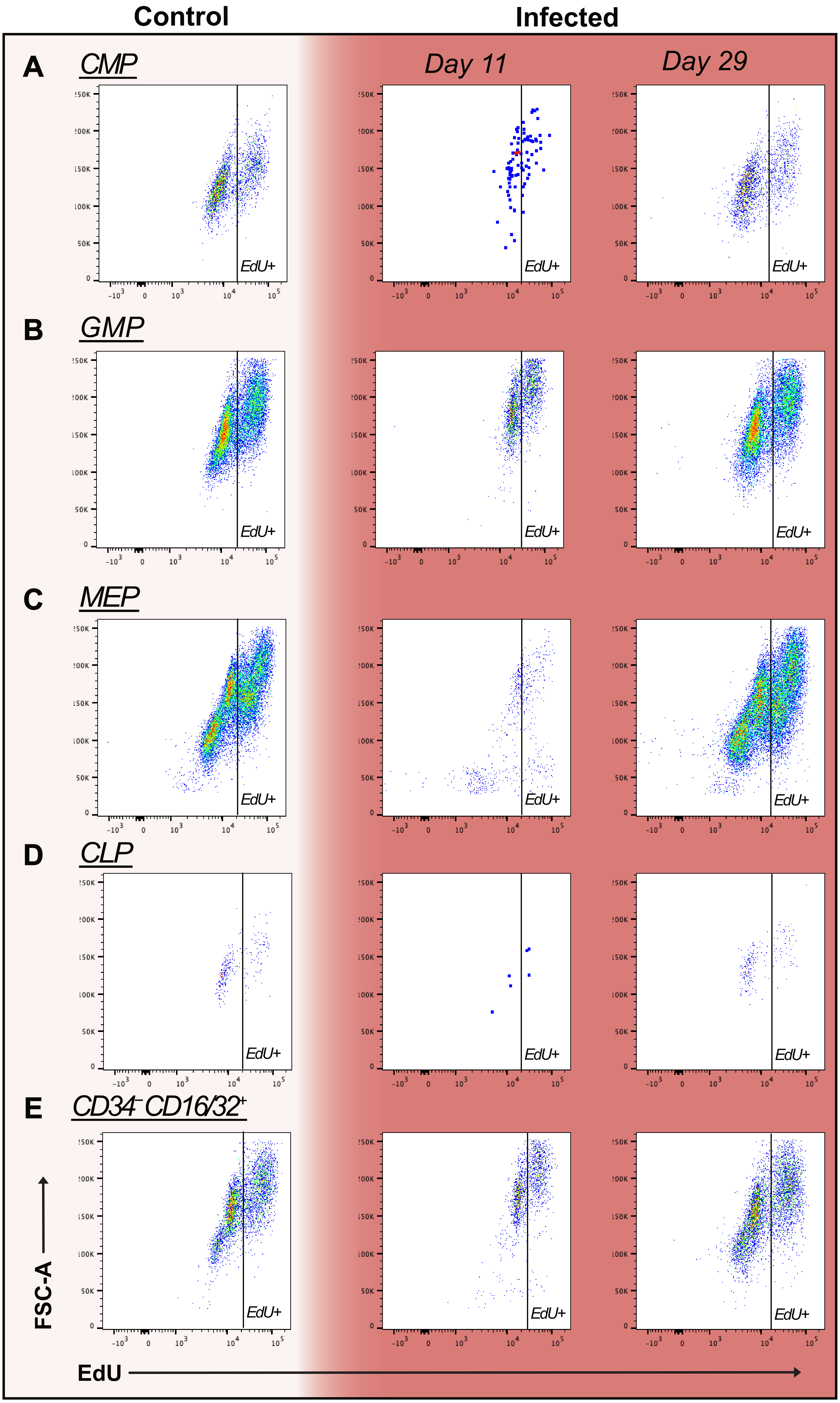

Figure S7. Proliferation of mature progenitor populations during *P. chabaudi* infection.

A-E. Representative flow cytometry plots showing in the S-phase of the cell cycle (EdU^{+}) within CMP (**A**), GMP (**B**), MEP (**C**), CLP (**D**) and CD34^{-}CD16/32^{+} (**E**) populations in controls and infected mice at day 11 and day 29 p.i.

Antibody	Clone	Manufacturer	Cat. no	Dilution
FITC anti-mouse CD4	RM4-5	Biolegend	100510	1/400
FITC anti-mouse CD5	53-7.3	Biolegend	100606	1/100
FITC anti-mouse CD8a	53-6.7	Biolegend	100706	1/200
FITC anti-mouse B220	RA3-6B2	Biolegend	103206	1/200
FITC anti-mouse Ter119	TER-119	Biolegend	116206	1/100
FITC anti-mouse Ly-6G/Ly-6C (Gr-1)	RB6-8C5	Biolegend	108406	1/400
APC/Cyanine7 anti-mouse CD117 (c-kit)	2B8	Biolegend	105826	1/200
PerCP/Cyanine5.5 anti-mouse Ly-6A/E (Sca-1)	D7	Biolegend	108124	1/300
Brilliant Violet 650 anti-mouse CD150	TC15-12F12.2	Biolegend	115931	1/200
PE/Cyanine7 anti-mouse CD48	HM48-1	Biolegend	103424	1/200
PE anti-mouse CD127	A7R34	Invitrogen	12-1271-82	1/100
FITC anti-mouse CD34	RAM34	Invitrogen	11-0341-81	1/50
APC anti-mouse CD135	A2F10	Biolegend	135310	1/200

Table S1. Details of antibodies used in this study.
